# Differential roles of species richness versus species asynchrony in regulating community stability along a precipitation gradient

**DOI:** 10.1002/ece3.5857

**Published:** 2019-11-21

**Authors:** Yonggang Chi, Zhuwen Xu, Lei Zhou, Qingpeng Yang, Shuxia Zheng, Shao‐peng Li

**Affiliations:** ^1^ College of Geography and Environmental Sciences Zhejiang Normal University Jinhua China; ^2^ State Key Laboratory of Vegetation and Environmental Change Institute of Botany Chinese Academy of Sciences Beijing China; ^3^ State Key Laboratory of Resources and Environmental Information System Institute of Geographic Sciences and Natural Resources Research Chinese Academy of Sciences Beijing China; ^4^ School of Ecology and Environment Inner Mongolia University Hohhot China; ^5^ Key Laboratory of Ecosystem Network Observation and Modeling Institute of Geographic Sciences and Natural Resources Research Chinese Academy of Sciences Beijing China; ^6^ Key Laboratory of Forest Ecology and Management Institute of Applied Ecology Chinese Academy of Sciences Shenyang China; ^7^ Zhejiang Tiantong Forest Ecosystem National Observation and Research Station School of Ecological and Environmental Sciences East China Normal University Shanghai China; ^8^ Institute of Eco‐Chongming (IEC) Shanghai China

**Keywords:** climate change, ecosystem functioning, primary productivity, semiarid ecosystem, species evenness, transect survey

## Abstract

Plant community may provide products and services to humans. However, patterns and drivers of community stability along a precipitation gradient remain unclear. A regional‐scale transect survey was conducted over a 3‐year period from 2013 to 2015, along a precipitation gradient from 275 to 555 mm and spanning 440 km in length from west to east in a temperate semiarid grassland of northern China, a central part of the Eurasian steppe. Our study provided regional‐scale evidence that the community stability increased with increasing precipitation in the semiarid ecosystem. The patterns of community stability along a precipitation gradient were ascribed to community composition and community dynamics, such as species richness and species asynchrony, rather than the abiotic effect of precipitation. Species richness regulated the temporal mean (*μ*) of aboveground net primary productivity (ANPP), while species asynchrony regulated the temporal standard deviation (*σ*) of ANPP, which in turn contributed to community stability. Our findings highlight the crucial role of community composition and community dynamics in regulating community stability under climate change.

## Graphical abstract

A regional‐scale transect survey was conducted over a 3‐year period from 2013 to 2015, along a precipitation gradient from 275 to 555 mm and spanning 440 km in length from west to east in a temperate semiarid grassland in northern China, a central part of the Eurasian steppe. Our study provided regional‐scale evidence that the community stability increased with increasing precipitation in the semiarid ecosystem.

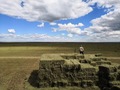

## 1 INTRODUCTION

Plant community may maintain biodiversity, conserve water resources, sequester carbon, and provide reliable products and services to humans (Li, Poisot, Waller, & Baiser, 2018; Oliver et al., 2015). However, the earth has been observed to be experiencing unprecedented climate change, such as global warming and intensive drought (Geng et al., 2019; Zhou, Wang, Chi, & Wang, 2018). Climate change would have profound effects on community composition and community dynamics (Chi, Xu, Shen, & Wang, 2013), and subsequently influence community stability (Shi et al., 2016; Zhou, Wang, Chi, Ju, et al., 2018). Meanwhile, plant community with higher stability is expected to have stronger capacity to resist disturbances and quicker speed to return to its original state after disturbances (Willis, Jeffers, & Tovar, 2018). Therefore, quantitative analysis of community stability is helpful for assessing ecosystem functioning and services under climate change.

Community stability is often defined as the constancy of the ecological variables over time and is usually described as the ratio of the temporal mean to the temporal standard deviation of aboveground net primary productivity (ANPP) (Lehman & Tilman, 2000). The temporal mean of ANPP represents the photosynthetic capacity of plant community (Huang et al., 2018). In comparison, the temporal *SD* of ANPP indicates the sensitivity of plant community to climate change (Craven et al., 2018). In theory, the different magnitudes in the changes of the temporal mean and the temporal *SD* of ANPP will contribute to a shift in the community stability (Shi et al., 2016). However, little is known about the ecological processes regulating the temporal mean and the temporal *SD* of ANPP.

Precipitation is an essential factor that largely controls community stability, especially in semiarid ecosystems (Huston, 2012). On the one hand, precipitation affects community stability (Wilcox, Blair, Smith, & Knapp, 2016) through the changes in abiotic drivers, such as soil water content (Knapp et al., 2008), temperature (Guo et al., 2015), incident radiation (Nijp et al., 2015), and soil nutrient availability (Niu et al., 2017). On the other hand, precipitation influences community stability via changes in community compositions and dynamics, such as species richness (Isbell, Polley, & Wilsey, 2009), species evenness (Hallett et al., 2014), and species asynchrony (Xu et al., 2015). Although the effects of precipitation on community stability have been well documented, the patterns of community stability along a precipitation gradient are inconsistent, varying from neutral (Hallett et al., 2014) to positive (Bai et al., 2008). Therefore, disentangling the relative contributions of the direct versus indirect effects of precipitation on the community stability is essential for gaining a thorough understanding of ecosystem functioning in semiarid regions under climate change.

**Figure 1:**
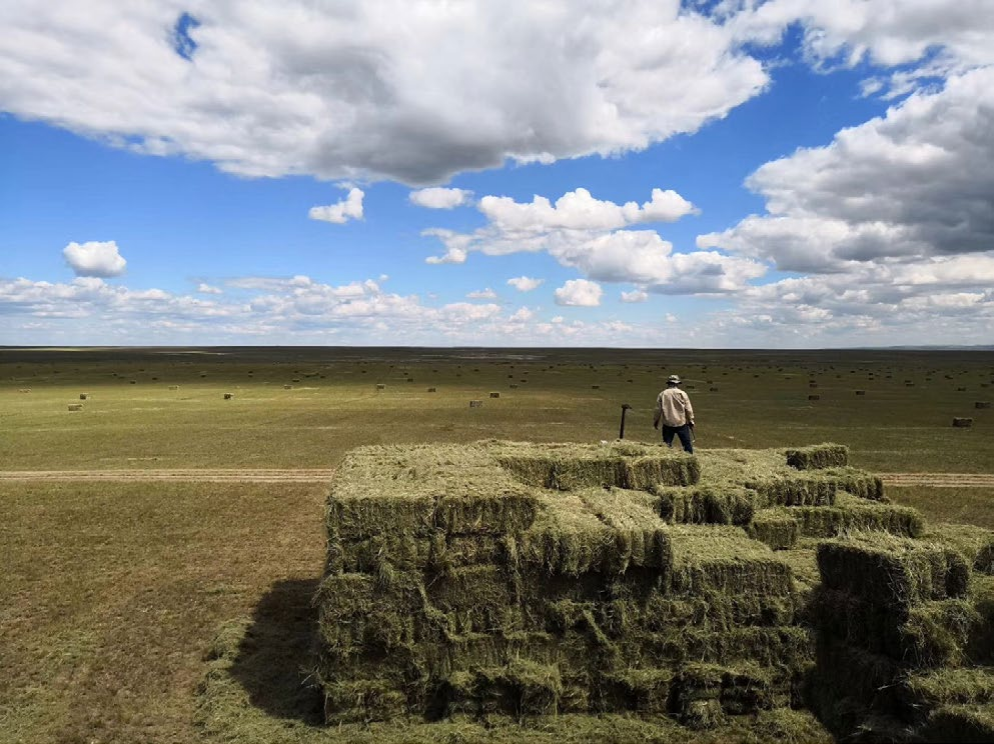
Herdsmen collected grass in East Ujimqin Banner (45.27°N, 116.72°E) on the temperate semiarid grassland in northern China, a central part of the Eurasian steppe

The semiarid grassland of northern China, with an area of 313 million hm^2^ and a plant species richness of 2,300 (Kang, Han, Zhang, & Sun, 2007), plays an important role in serving the economy and well‐being of people residing in the region (Figure 1). The Inner Mongolian grasslands are now fenced off into individual pastures for two different management forms (mowing and grazing) (Chi, Zhou, Yang, Li, & Zheng, 2019). The grass for mowing is cut by machines and compressed into hay bales that can be delivered to a herder's home. Herdsmen no longer need to find good pastures and have said goodbye to the traditional nomadic way. In addition, a decrease in precipitation amount is observed in this area under climate change (Xu et al., 2012). Drought is likely to cause deterioration in ecosystem functioning and services provided by grasslands (Chi et al., 2018). It is necessary to evaluate the community stability of this region under precipitation change.

In this study, a regional‐scale transect survey was conducted over a 3‐year period from 2013 to 2015, along a precipitation gradient from 275 to 555 mm and spanning 440 km in length from west to east in a temperate semiarid grassland of northern China, a central part of the Eurasian steppe. We aimed to (a) explore the patterns of community stability along a precipitation gradient and (b) elucidate the drivers of community stability at the regional scale. To address the two questions, we hypothesize that drier sites have a lower ANPP, species richness, and community stability, while wetter sites have a higher ANPP, species richness, and community stability, because plant growth and community composition are regulated predominantly by precipitation in semiarid grassland.

## 2 MATERIALS AND METHODS

### 2.1 Study area and experimental design

**Figure 2:**
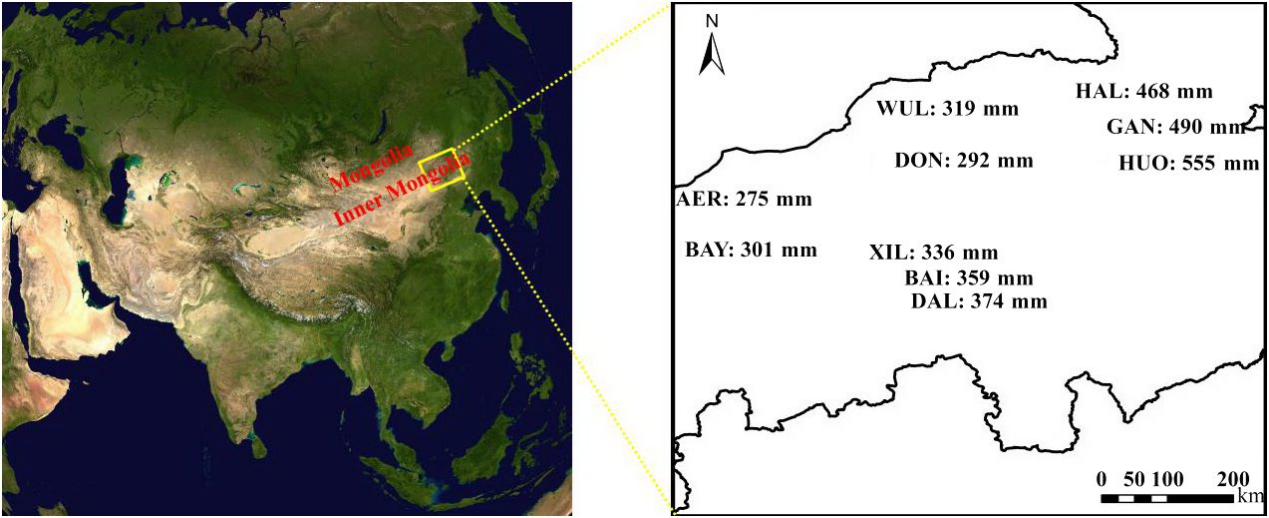
Study area and locations of the 10 sites along a precipitation gradient from 275 to 555 mm and spanning 440 km in length from west to east in temperate semiarid grassland in northern China. The satellite image of Asia is from the National Aeronautics and Space Administration. The mean annual precipitation (MAP) in each site is indicated next to the circle. Individual site name abbreviations are as follows: AER, Aershan; BAI, Baiyinxile; BAY, Bayanbaolige; DAL, Dalinuoer; DON, Dongwu; GAN, Ganqiaobao; HAL, Halagaitu; HUO, Huolinguole; WUL, Wulagai; XIL, Xilinhot

The study was conducted in temperate semiarid grassland in northern China (Figure 2). The study area is located at 43.46°N–45.83°N, 115.78°E–119.72°E, and the elevation is between 908 and 1,257 m. The long‐term (1980–2015) mean annual precipitation (MAP) ranges from 275 mm for the drier western regions to 555 mm in the wetter eastern regions, approximately 80% of which falls during the growing season from May to August. The mean annual temperature (MAT) ranges from 0.02 to 2.27°C, with the lowest mean monthly temperature in January and the highest in July.

A regional‐scale transect was established along a precipitation gradient, which spanned 440 km in length from west to east (Table S1). A total of 10 sites were selected along this transect, including 6 typical steppe and 4 meadow steppe sites. The selected area was not grazed by large herbivores but mowed for hay harvest in the autumn. Land use and management were consistent across the ten sites. The typical steppe, located in the western part of the precipitation gradient, is dominated by *Leymus chinensis* (Trin.) Tzvel. (Graminoid), *Carex enervis* C. A. Mey. (Forbs), *Cleistogenes squarrosa* (Trin.) Keng (Graminoid), and *Stipa grandis* P. Smirn. (Graminoid), has lower species richness and productivity. The meadow steppe, located in the eastern part of the precipitation gradient, is dominated by *C. enervis* C. A. Mey. (Forbs), *L. chinensis* (Trin.) Tzvel. (Graminoid), *C. squarrosa* (Trin.) Keng (Graminoid), and *Agropyron cristatum* (L.) Garrtn. (Graminoid), has higher species richness and productivity. A total of 99 species were found in the study area. However, not all species appeared across both types of steppe. For example, *Artemisia tanacetigolia* L. (Forbs) was found only in the typical steppe, while *Iris ventricosa* Pall. (Forbs) was found only in the meadow steppe. The precipitation range of typical steppe is 250–400 mm, while that of meadow steppe is 350–500 mm (Bai et al., 2008). Therefore, the 350–400 mm precipitation occurs in the boundary between typical steppe and meadow steppe. In our current study, the main plant functional type in the sites AER, DON, BAY, WUL, XIL, and BAI are typical steppe, while the sites HAL, GAN, HUO, and DAL are situated in meadow steppe.

The soil in typical steppe and meadow steppe is classified as typical chestnut and dark chestnut, respectively, both of which are Calcic Chernozem according to International Society of Soil Science (Chi, Xu, Shen, Yang, et al., 2013). Soil pH in the top 10 cm layer decreased from 7.43 ± 0.04 in the typical steppe to 7.03 ± 0.06 in the meadow steppe (*p* < .001, Table S1). However, no statistical difference in soil total nitrogen of the top 10 cm layer was observed for the typical steppe (1.26 ± 0.08 g/kg) and the meadow steppe (1.21 ± 0.09 g/kg) (*p* = .683, Table S1).

### 2.2 Transect survey and field sampling

A transect survey was carried out during August, corresponding to the annual peak in standing biomass, over a 3‐year period from 2013 to 2015. The selected area in each site was relatively uniform based on plant height and community composition. The 1 × 1 m quadrats were selected within a 100 × 100 m area on each study site in 2013. The distance between any two quadrats was more than 10 m. The quadrats of 2014 and 2015 were selected next to the quadrats of 2013 in order to reduce spatial heterogeneity of plant community. Species richness was recorded according to the total number of plant species found in each 1 × 1 m quadrat. The number of individuals of each species in each quadrat was counted. Then, all vascular plants within each 1 × 1 m quadrat were clipped and sorted into species. The aboveground biomass of each species in each quadrat was determined as the weight of the aboveground plant material after oven drying at 65ºC for 48 hr. The annual ANPP of the community was the sum of the aboveground biomass of each species, which is an acceptable approximation in this region because aboveground plant tissues die during winter (Zhang et al., 2018). A total of 180 quadrats were sampled (i.e., 6 quadrats per site × 10 sites per year × 3 years = 180 quadrats).

### 2.3 Community stability and dynamics

The community stability was quantified as the ratio of the temporal mean (*μ*) to the temporal *SD* (*σ*) of ANPP over the 3‐year period of the transect survey (Tilman, 1999). The temporal mean (*μ*) of ANPP was defined as the mean of each individual quadrat over 3 years. A larger value of community stability indicates smaller interannual variability in community biomass (Hautier et al., 2014).

Species richness (*S*) was defined as the total number of plant species detected in the quadrat. Species evenness (*E*) was quantified by Pielou's evenness index (Pielou, 1966), defined as:

(1) $$E=-\sum_{i=1}^{S}\left(P_{i}\ln P_{i}\right)/\ln S$$

where *S* is the number of species, and *P_i_* is the relative abundance of the *i*th species. This index is 1 when the relative abundances of each species are perfectly equal and 0 when the relative abundances of each species are perfectly unequal. Species asynchrony was quantified by the community‐wide asynchrony index (Loreau & de Mazancourt, 2008), defined as:

(2) $$1-\varphi_{x}=1-\sigma^{2}/{\left(\sum_{i=1}^{S}\sigma_{i}\right)}^{2}$$

where *φ_x_* is species synchrony, *σ* ^2^ is the temporal variance of the annual ANPP, and *σ_i_* is the *SD* of the annual ANPP of the *i*th species in a plot with *S* species. This index is 1 when the interannual changes of species are perfectly asynchronized and 0 when the interannual changes of species are perfectly synchronized.

### 2.4 Climate data

The MAP and MAT of each site from 1980 to 2015 were interpolated with a geographic information system (GIS)‐based multiple‐regression method using latitude, longitude, and altitude as predictors. Meteorological data from 47 meteorology stations in the temperate semiarid grassland of northern China were provided by the Chinese National Meteorological Information Centre.

### 2.5 Statistical analysis

Linear mixed models were used to evaluate the effects of MAP, MAT, elevation, vegetation type and site on the community stability, species richness, and species asynchrony. In all models, MAP, MAT, elevation, and vegetation type served as fixed factors, and site was included as a random factor. The data of community stability were natural log transformed to meet the assumptions of normality and heterogeneity in linear mixed models. In addition, linear regression analyses were used to evaluate the effects of MAP, species richness, and species asynchrony on the community stability. A probability (*p*) value of <.05 was considered to be statistically significant. All statistical analyses were performed using SPSS 17.0 (SPSS Inc.).

Structural equation models (SEMs) were used to explore the direct effects of precipitation on the community stability versus the indirect effects through changes in species richness and species asynchrony. We focused on the two components of community stability (the temporal mean and the *SD* of ANPP) as our two response variables, to disentangle whether precipitation influenced community stability via changes in temporal mean and/or temporal variation. The temporal mean is a proxy for the level of ANPP, while the temporal *SD* is a proxy for the annual variation in ANPP. The two components of community stability are related to forage provision, forage store, economy, and well‐being of people residing in the region. The fit of the model was evaluated using the chi‐squared (*χ* ^2^) test and the root mean square error of approximation (RMSEA). SEMs analysis was performed with Amos 21.0 (Amos Development Corporation).

Redundancy analysis (RDA) was performed to evaluate community composition and dynamics shifted across sites and across years. Climatic, topographic, and soil properties were included in this analysis such as MAP, MAT, elevation, and soil pH. Environmental factors with significant influence on community structure were selected based on the results of forward selection. RDA was applied using Canoco Version 5.0 (CANOCO).

## 3 RESULTS

### 3.1 Community stability and its drivers

**Figure 3:**
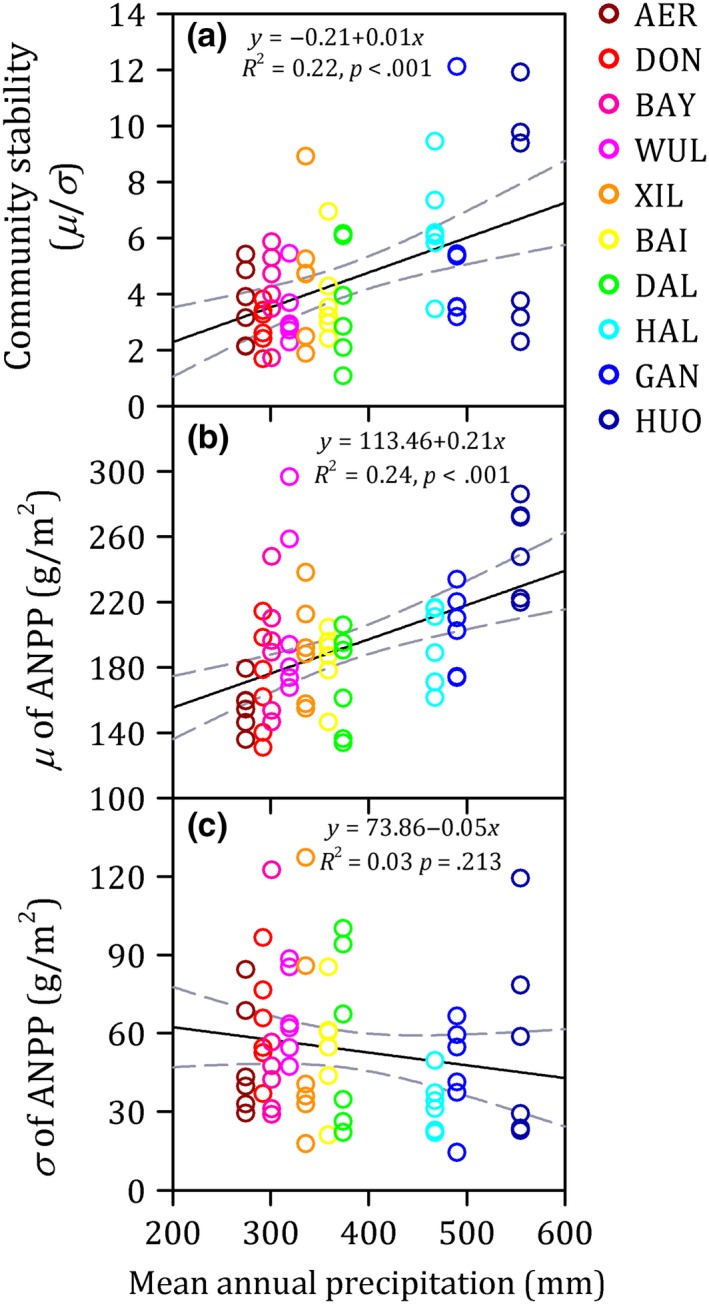
The relationships between mean annual precipitation (MAP) and community stability (a), the temporal mean (*μ*) of aboveground net primary productivity (ANPP) (b), and the temporal *SD* (*σ*) of ANPP (c) along a precipitation gradient from 275 to 555 mm and spanning 440 km in length from west to east in a temperate semiarid grassland in northern China. The gray lines show the linear fit within the 95% confidence interval. Each color corresponds to one site

The results produced by the linear mixed models indicated that MAP was a significant explanatory variable of community stability and species richness (both *p* < .05), while MAT, elevation, and vegetation type were not significant factors (all *p* > .05) (Table S2). Community stability increased significantly from the drier to the wetter regions across a precipitation gradient (*R* ^2^ = 0.22, *p* < .001; Figure 3). The temporal mean (*μ*) of ANPP was significantly positively related with MAP (*R* ^2^ = 0.24, *p* < .001; Figure 3). In contrast, the temporal *SD* (*σ*) of ANPP was not significantly related with MAP, but showed a general decreasing trend from the drier to the wetter sites (*R* ^2^ = 0.03, *p* = .213; Figure 3).

**Figure 4:**
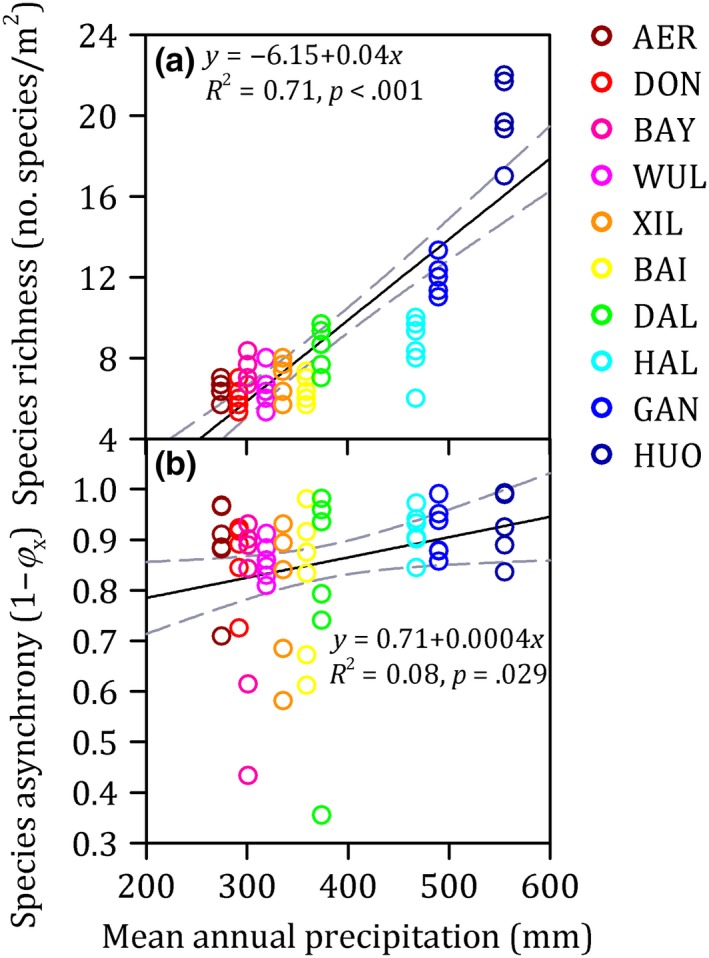
The relationships between mean annual precipitation (MAP) and species richness (a) and species asynchrony (b) along a precipitation gradient from 275 to 555 mm and spanning 440 km in length from west to east in temperate semiarid grassland of northern China. The gray lines show the linear fit within the 95% confidence interval. Each color corresponds to one site

**Figure 5:**
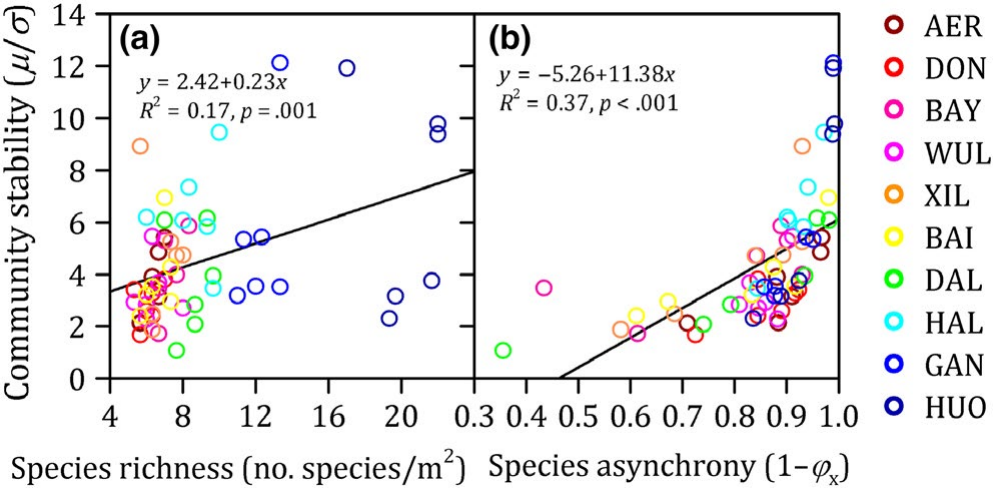
Community stability as a function of species richness (a) and species asynchrony (b) along a precipitation gradient from 275 to 555 mm and spanning 440 km in length from west to east in a temperate semiarid grassland in northern China. Each color corresponds to one site

Species richness and species asynchrony increased significantly from the drier to the wetter regions across a precipitation gradient (*R* ^2^ > 0.08 and *p* < .05 for both, Figure 4). Community stability was significantly positively related with species richness (*R* ^2^ = 0.17 and *p* = .001) and species asynchrony (*R* ^2^ = 0.37 and *p* < .001) (Figure 5). In addition, correlation analyses were performed to evaluate the relationships of species richness with species evenness (Figure S1). Species richness was significantly positively correlated with species evenness (*p* < .001, Figure S1). So, species richness was maintained as explanatory variable, while species evenness was removed.

### 3.2 Direct and indirect effects on community stability

**Figure 6:**
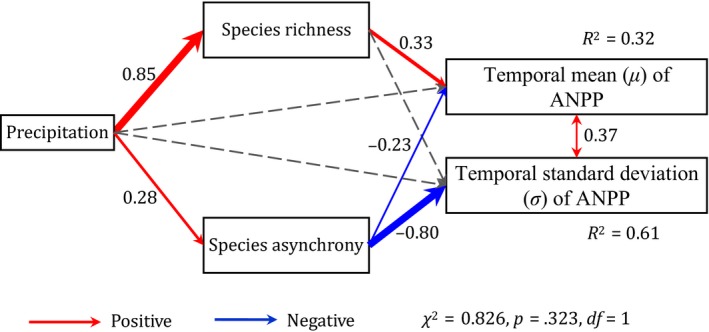
Structural equation models (SEMs) were used to examine the effects of precipitation on the two components of the community stability, the temporal mean (*μ*) of aboveground net primary productivity (ANPP), and the temporal *SD* (*σ*) of ANPP, via pathways of direct versus indirect effects (*n* = 60, 6 quadrats per site × 10 sites). The solid red arrows represent positive paths (*p < *.05), the solid blue arrows represent negative paths (*p < *.05), and the dotted gray arrows represent nonsignificant paths (*p > *.05). Values associated with the solid arrows represent standardized path coefficients, that is, partial regression coefficients. The arrow width is proportional to the strength of the relationship. The amount of variance explained (*R* ^2^) in the two components (*μ* and *σ* of ANPP) of community stability is also shown

**Figure 7:**
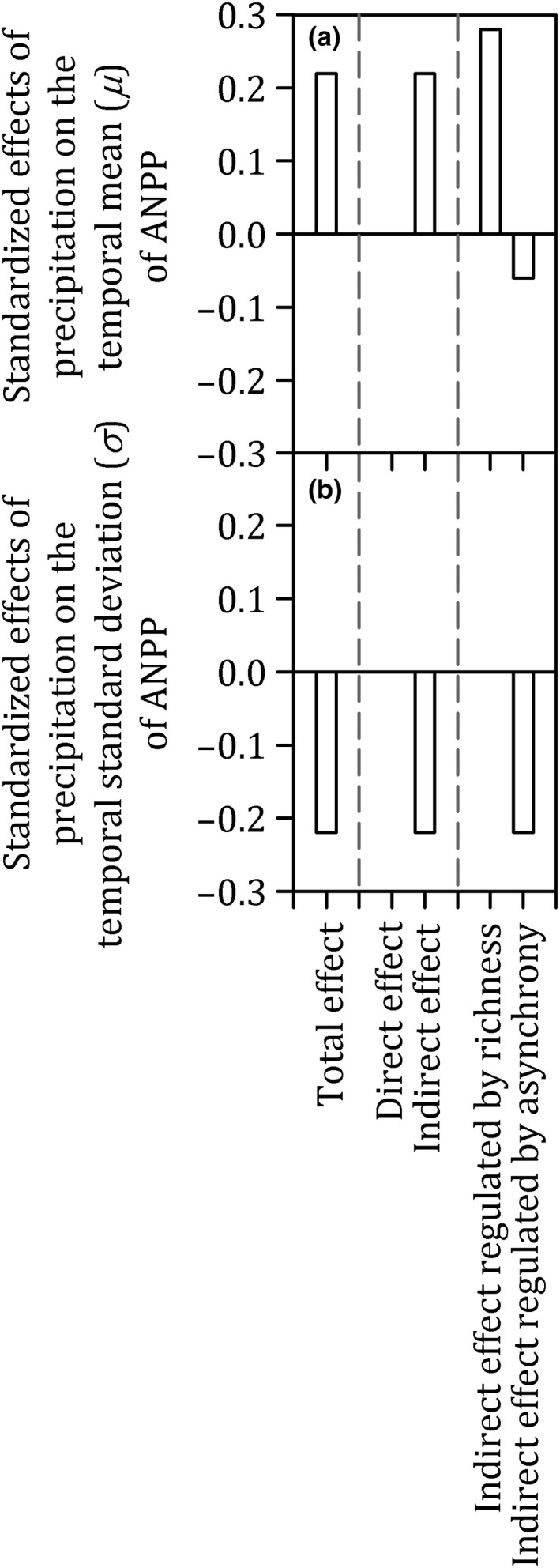
Standardized effects of precipitation on the (a) the temporal mean (*μ*) of community biomass, and (b) the temporal *SD* (*σ*) of aboveground net primary productivity (ANPP), derived from the structural equation models (SEMs) of Figure 6

The SEMs showed that precipitation had no direct effect on *μ* of ANPP (SEMs; path coefficients = 0.28, but *p* = .171) or *σ* of ANPP (SEMs; path coefficients = −0.08, but *p* = .611), but it had an indirect effect via community composition and dynamics (Figures 6 and 7). Specifically, precipitation increased not only species richness (SEMs; path coefficients = 0.85 and *p* < .001), which in turn increased *μ* of ANPP (SEMs; path coefficients = 0.33 and *p* = .046), but also species asynchrony (SEMs; path coefficients = 0.28 and *p* = .023), which in turn decreased *μ* of ANPP (SEMs; path coefficients = −0.23 and *p* = .041; Figure 6). Because the positive effect via species richness (0.85 × 0.33 = 0.28) was greater in magnitude than the negative effect via species asynchrony (0.28 × −0.23=−0.06), the total effect of precipitation on *μ* of ANPP was positive (0.28 – 0.06 = 0.22; Figure 7). Conversely, precipitation increased species asynchrony (SEMs; path coefficients = 0.28 and *p* = .023), which in turn decreased *σ* of ANPP (SEMs; path coefficients = −0.80 and *p < *.001; Figure 6). Therefore, the total effect of precipitation on *σ* of ANPP was negative (0.28 × −0.80 = −0.22; Figure 7). When the two stability components, that is, *μ* and *σ* of ANPP, were evaluated together, the results suggest that the overall effect of precipitation on the community stability is positive (Figures 3 and 6).

**Figure 8:**
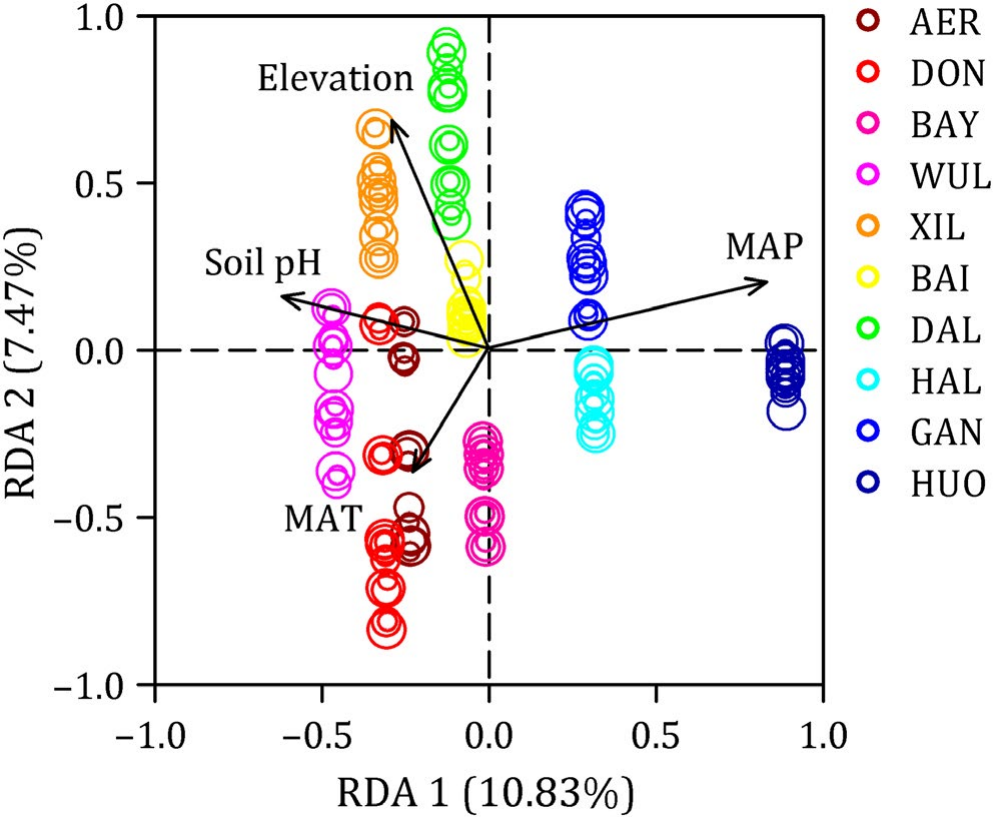
Redundancy analysis (RDA) correlation plot showing variance in plant community composition across the ten sites explained by significant environmental variables (*p* < .05). Each color corresponds to one site. Sampling years are distinguished by circle sizes as following: 2013 (small), 2014 (medium), and 2015 (large). MAP, mean annual precipitation; MAT, mean annual temperature

The RDA showed that four environmental factors (MAP, MAT, elevation, and soil pH) were selected and included in the RDA framework (Figure 8). The RDA was globally significant (*p* = .006) with an adjusted coefficient of determination (*R*_adj_^2^) of 0.072. The first two axes of the RDA model explained 10.83% and 7.47% of the total variation, respectively (Figure 8). RDA correlation plot showed variance in plant community composition across the ten sites explained by significant environmental variables (*p* < .05, Figure 8).

## 4 DISCUSSION

### 4.1 Patterns and drivers of community stability along a precipitation gradient

Our study showed that community stability increased from the drier to the wetter regions in the temperate semiarid grassland of northern China, a central part of the Eurasian steppe (Figure 3). The results of our study from the transect survey at the regional scale confirmed the positive precipitation–stability relationships reported from the manipulation experiments at the local scale (Xu et al., 2015). Observational study predicts a decrease in annual precipitation amount in this area (Xu et al., 2012). Therefore, grassland community stability may be weakened in the future, providing lesser predictability of ecosystem functioning and services.

In the current study, the patterns of community stability along a precipitation gradient were ascribed to community composition and dynamics, such as species richness and species asynchrony, rather than the abiotic effect of precipitation (Figure 5). Although a few papers have reported that abiotic factors may directly influence the community stability (Bai et al., 2008), many studies found that community composition and dynamics were the dominant determinants that indirectly affected the variation in community stability under climate change (Hautier et al., 2014). For example, experimental warming lowered community stability by reducing the degree of species asynchrony on the Tibetan Plateau (Ma et al., 2017). An increase in precipitation elevated the community stability by increasing the stability of the dominant species in semiarid grassland (Xu et al., 2015). Nitrogen enrichment weakened community stability by decreasing the number of plant species on the Eurasian steppe (Lan & Bai, 2012). Our results based on transect survey highlight the importance of community composition and dynamics in regulating community stability under climate change. Biodiversity and their asynchronous responses might provide insurance effects for ecosystem functioning and services in Eurasian steppe.

### 4.2 Differential drive mechanisms of species diversity versus species asynchrony

Although both species richness and species asynchrony drove the change in the community stability along a precipitation gradient, the two factors governed different components of community stability (Figures 6 and 7). In principle, species richness may influence community stability because more diverse communities are more likely to contain species that are resistant to climate change (Loreau & Hector, 2001; Tilman et al., 2001). Species asynchrony can mirror interspecific competition and heterogeneity in species responses to climate change (response diversity) (Blüthgen et al., 2016). Our study based on the transect survey further quantified that species richness positively regulated the temporal mean (*μ*) of ANPP, whereas species asynchrony negatively controlled the temporal *SD* (*σ*) of ANPP (Figure 6). Furthermore, a positive relationship between species richness and *μ* of ANPP has been reported from theoretical model (Mougi & Kondoh, 2012), field observation (Bai et al., 2008), and a manipulative experiment (Gross et al., 2014). A negative relationship between species asynchrony and *σ* of ANPP has also been found in a meta‐analysis of 39 grassland biodiversity experiments (Craven et al., 2018). Therefore, our study demonstrated the differential mechanisms of species richness versus species asynchrony in regulating community stability.

## 5 CONCLUSIONS

Our study found that community stability increased with increasing precipitation in the temperate semiarid grassland. This result suggested that a decrease in annual precipitation amount under climate change could reduce the ability of our study community to provide reliable products and services for humans. Furthermore, our study provided the evidence that species richness regulated *μ* of ANPP, while species asynchrony regulated *σ* of ANPP. These findings highlight the relationship between community composition and dynamics and ANPP in semiarid ecosystems under climate change.

## CONFLICT OF INTERESTS

The authors have no conflict of interests to declare.

## AUTHOR CONTRIBUTIONS

Yonggang Chi (YC) designed the research. YC and Lei Zhou (LZ) conducted the field sampling and the sample analyses. YC, Zhuwen Xu (ZX), LZ, Qingpeng Yang (QY), Shuxia Zheng (SZ), and Shao‐peng Li (SL) analyzed the data and wrote the manuscript. All authors approved the final version of the manuscript.

## Data Availability

Data available from the Dryad Digital Repository: https://doi.org/10.5061/dryad.qz612jm96.
