# Supplementary material for: Differential roles of species richness versus species asynchrony in regulating community stability along a precipitation gradient
Source: Ecol Evol. 2019 Nov 21;9(24):14244–52. doi: 10.1002/ece3.5857 (PMC6953564; doi:10.1002/ece3.5857)
Supplement: Supplementary file 1 [file ECE3-9-14244-s001.docx]

**Supporting Information**

**Table S1** Summary of 10 sites in the temperate steppe in Inner Mongolia, China. MAP, mean annual precipitation; CV_precipitation_, inter-annual variance of precipitation; MAT, mean annual temperature; TSN, total soil nitrogen.

| Experimental sites | Abbrev. | Latitude (N) | Longitude (E) | Elevation (m) | MAP (mm) | CV_precipitation_ (%) | MAT (℃) |  | Soil pH | TSN (g/kg) | Vegetation type |
| --- | --- | --- | --- | --- | --- | --- | --- | --- | --- | --- | --- |
| Aershan | AER | 44.73 | 115.78 | 994 | 274.53 | 23.4 | 1.93 |  | 7.50 | 0.56 | Typical steppe |
| Dongwu | DON | 45.27 | 116.72 | 931 | 292.19 | 22.7 | 1.29 |  | 7.35 | 1.87 | Typical steppe |
| Bayanbaolige | BAY | 44.05 | 115.94 | 1022 | 301.07 | 23.4 | 2.27 |  | 7.56 | 1.65 | Typical steppe |
| Wulagai | WUL | 45.68 | 117.46 | 965 | 319.16 | 22.3 | 0.40 |  | 7.27 | 1.18 | Typical steppe |
| Xilinhot | XIL | 43.93 | 116.28 | 1230 | 335.76 | 21.4 | 0.98 |  | 7.41 | 1.38 | Typical steppe |
| Baiyinxile | BAI | 43.62 | 116.65 | 1187 | 358.73 | 19.3 | 1.37 |  | 7.22 | 0.91 | Typical steppe |
| Dalinuoer | DAL | 43.46 | 116.75 | 1257 | 373.72 | 18.1 | 1.09 |  | 7.48 | 0.66 | Meadow steppe |
| Halagaitu | HAL | 45.83 | 119.43 | 908 | 467.52 | 23.9 | 0.34 |  | 6.92 | 1.67 | Meadow steppe |
| Ganqiaobao | GAN | 45.69 | 119.52 | 980 | 489.81 | 23.9 | 0.02 |  | 6.96 | 0.96 | Meadow steppe |
| Huolinguole | HUO | 45.43 | 119.72 | 960 | 554.81 | 24.5 | 0.99 |  | 6.77 | 1.53 | Meadow steppe |

**Table S2** Linear mixed models for the effects of mean annual precipitation (MAP), mean annual temperature (MAT), elevation, Vegetation type and site on the plant communities at the regional scale in the grassland. Coefficient values are the estimated standardized coefficients. SE and *p* value are the standard error and significance, respectively.

| Coefficients | Community stability | | |  | Species richness | | |  | Species asynchrony | | |
| --- | --- | --- | --- | --- | --- | --- | --- | --- | --- | --- | --- |
|  | Value | SE | *p* |  | Value | SE | *p* |  | Value | SE | *p* |
| Intercept | -1.118 | 4.432 | 0.802 |  | -13.081 | 10.893 | 0.284 |  | **1.017** | **0.248** | **0.001** |
| MAP | **0.015** | **0.007** | **0.023** |  | **0.056** | **0.016** | **0.018** |  | 0.001 | 0.001 | 0.449 |
| MAT | 0.310 | 0.546 | 0.573 |  | 2.347 | 1.343 | 0.141 |  | -0.016 | 0.031 | 0.603 |
| Elevation | -0.001 | 0.002 | 0.772 |  | -0.002 | 0.006 | 0.727 |  | -0.001 | 0.001 | 0.079 |
| Vegetation type | 0.375 | 1.162 | 0.748 |  | 1.300 | 2.855 | 0.455 |  | 0.004 | 0.065 | 0.946 |

**

**

**Figure S1** The relationships between species richness and species evenness across the ten sites along a precipitation gradient from 275 to 555 mm and spanning 440 km in length from west to east in temperate semi-arid grassland in northern China. Each color corresponds to one site.
